# Effects of Caffeine Ingestion on Morning Cognitive and Muscle Strength Measures in Males: A Standardized Approach

**DOI:** 10.3390/nu18060954

**Published:** 2026-03-18

**Authors:** João P. S. Agulhari, Neil Chester, Magali Giacomoni, Karl C. Gibbons, Dani Hajdukiewicz, Haydyn L. O’Brien, Thomas D. O’Brien, Jack Jensen, Briony Lucas, Samantha L. Moss, Samuel A. Pullinger, Ben J. Edwards

**Affiliations:** 1 Research Institute for Sport and Exercise Sciences, Liverpool John Moores University, Liverpool L3 5AF, UK; j.agulhari@2022.ljmu.ac.uk (J.P.S.A.); n.chester@ljmu.ac.uk (N.C.); k.c.gibbon@ljmu.ac.uk (K.C.G.); d.hajdukiewicz@ljmu.ac.uk (D.H.); h.obrien@2023.ljmu.ac.uk (H.L.O.); t.d.obrien@ljmu.ac.uk (T.D.O.); j.jensen@ljmu.ac.uk (J.J.); b.lucas@ljmu.ac.uk (B.L.); s.l.moss@ljmu.ac.uk (S.L.M.); samuelpullinger@inspireinstituteofsport.com (S.A.P.); 2 Unit of Training and Research Department, Subject Area Sciences and Techniques of Physical and Sports Activities, Laboratory Youth Physical Activity Sport and Health (J-AP2S), University of Toulon, 83130 La Garde, France; magaligiacomoni@univ.tlnfr; 3 Sport Science Department, Inspire Institute of Sport, Vidyanagar, Bellary 583275, India

**Keywords:** bench press, back squat, executive function, diurnal variation, isometric maximum voluntary contraction

## Abstract

**Background/Objectives**: We investigated whether ingestion of caffeine (~1 h before) was beneficial to subsequent morning (07:30 h), mood, strength and cognitive measures. **Methods**: Fourteen recreationally active males were recruited and completed six sessions: (i) one repetition maximum (1RM) for bench press and back squat; (ii) two familiarization sessions of strength measures; (iv) three experimental conditions administered in a double-blinded, randomized counterbalanced design order, either caffeine (Caffeine [CAFF], 300 mg or 2.8–4.3 mg/kg body weight), placebo (Placebo [PLAC]) ingested at 06:30 h, or no-pill control (No Pill [NoPill]). For each experimental session, on arrival at the laboratory, rectal and skin temperature were measured as well as a battery of cognitive performance through a battery of tests (trail-making test, Rey’s auditory verbal learning test, and Stroop word–colour interference test). Thereafter, maximum voluntary contraction on an isometric chair (MVC) without and with stimulation was conducted, and three repetitions were performed at 40, 60 and 80% of 1RM for bench press and back squat. Average power (AP), average velocity (AV), peak velocity (PV), mean propulsive velocity (MPV), average acceleration (RDV), displacement (D) and time-to-peak velocity (tPV) were recorded using MuscleLab linear encoders. Rating of perceived exertion and effort was asked after each set (RPE). The data was analysed using a general linear model with repeated measures. **Results**: MVC peak-force values with and without stimulation showed a significant increase in the CAFF condition compared to values for NoPill and with stimulation PLAC conditions (stim: Δ9.0 and 8.7%; no stim: 8.3%; *p* < 0.05; η^2^_p_ = 0.33 and 0.42). Greater muscle % activation was achieved for the CAFF than the other conditions (~6%, *p* ≤ 0.042; η^2^_p_ = 0.33). In the non-stimulated MVC, RPE was perceived as easier (4.8%, *p* = 0.04). AV and MPV values were higher in both bench press (Δ3.3 and 4.6%) and back squat (Δ7.7 and 9.2%) in CAFF than the PLAC condition (*p* = 0.031; η^2^_p_ = 0.24 and 0.23 and 0.24 and 0.32). CAFF improved auditory total recall compared to NoPill (9.5%, *p* = 0.040; η^2^_p_ = 0.22). **Conclusions**: Early morning ingestion of caffeine improved MVC to levels observed by others in the evening, as well as some aspects of bench press, back squat and recall performance. Caffeine ingestion had no effect on core temperature, mood, tiredness, alertness or other measures of cognitive performance.

## 1. Introduction

Tournament scheduling and environmental factors often require athletes to compete in the morning [1]. Equally due to time constraints, athletes may choose to train in the morning [2]. However, muscle power output and force production are higher in the mid-afternoon or early evening, whatever the method used, the muscle groups tested or the mode and speed of contraction [3]. Cognitive performance is another essential determinant for many athletes that has been shown to be time-of-day- and variable-dependent [4]. Considered as multifactorial, it includes many different components related to attention, accuracy, consistency, reaction time, vigilance, decision making and executive functions [4]. Executive functions notably include the ability to plan and coordinate considered action while updating it with inhibition processes of distractions to focus attention on the relevant information. Subjective negative mood states (fatigue) and motivation levels, which are essential elements for tasks requiring higher cognitive function, are poorer in the morning than the evening [2].

Caffeine (1,3,7-trimethylxanthine) is amongst the most extensively researched ergogenic supplements within the field of sport, aimed at enhancing alertness, concentration, and cognitive and physical performance [5,6]. Caffeine’s primary mechanism of action in the brain is the blockage of adenosine receptors, which play a central role in the regulation of the sleep–wake cycle by increasing sleep pressure throughout the activity phase. It is thought that by blocking these receptors, caffeine counteracts natural tiredness, improving attention, focus and overall cognitive performance. Michell and Redman [7], one of the few studies to investigate caffeine effects on morning cognitive performance, reported an improvement in a simple search task of 2.5% in undergraduate students when 4 mg·kg^−1^ body mass of caffeine was consumed 1 h before a 07:00 h experimental session versus a placebo. Unlike the sparce research investigating the cognitive effects of morning caffeine ingestion, its effects on muscle performance have been demonstrated elsewhere (for a review, see [8]). Caffeine (3 mg·kg^−1^ body mass) ingested 60 min before exercise increased dynamic strength and power output of upper and lower muscle groups in the morning (10:00 h) in resistance-trained men, with 4.6–5.3% improvement compared to a placebo [9]. Similarly, caffeine (3 mg·kg^−1^ body mass) ingested 60 min before exercise at 09:00 h improved muscular strength/power at moderate-to-high loads (75–90% 1RM) and endurance performance (65% 1RM) in the back squat while counteracting morning declines at light load (25% 1RM) for both back squat and bench press without altering electrical activity [10]. These benefits are likely due to increased neural activation, enhanced calcium release in muscles, and a reduction in the perception of effort during exercise, making it easier to exert maximal effort [11,12,13].

The observation of caffeine’s ability to improve morning cognitive and physical performance may be obscured by lack of rigour and standardization in the method employed (such as timing of the ingestion and dose chosen) [8]. Many studies do not report any control condition that considers a placebo effect (NoPill) [6] or familiarization with the tests to be conducted [14], recruit the sample size based on a power calculation and do not employ standardization of participants’ habitual caffeine use (low, medium or high caffeine daily users) [7]. In addition, studies have often failed to control important factors such as chronotype, time of year, time of day and participants’ quality of sleep, which specifically relates to investigations of chronobiological nature and other considerations [14,15,16]. The main biases in individual studies come from “bias in measurement of the outcome” and “bias in selection of the reported result”, with a lack of well-controlled studies [17]. Recent systematic reviews highlight a rigorous approach that needs to be adopted by future investigations regarding measuring possible daily variations in human exercise performance [18,19].

Therefore, the aims of this study were to use a well-controlled and scientifically thorough protocol to assess the effect of 300 mg of caffeine (CAFF) vs. a placebo (PLAC) vs. no pill (NoPill) on morning (a) strength and power output measured via the Biodex Isometric MVC as well as the Muscle Lab force–velocity linear encoder [such as peak force (PF), % muscle activation, average power (AP), average velocity (AV), peak velocity (PV), mean propulsive velocity (MPV), rate of development of velocity (RDV), displacement (D) and time to peak velocity (tPV)] and (b) cognitive performance (including tasks of attention, memory and executive function). We chose a population of low daily users, <150 mg, to reduce effects of caffeine withdrawal symptoms in the non-caffeine condition on performance, as well as maximizing caffeine effects at the 300 mg dose administered. A secondary aim was to measure core body and skin temperature, mood and subjective perceived effort and exertion (RPE). We hypothesized that caffeine would improve morning strength and cognitive measures compared to the other conditions (no pill and placebo) whilst reducing RPE.

## 2. Materials and Methods

### 2.1. Participants

Fourteen male adults of biological sex and gender who were classified as “recreationally active” by the “Participant classification framework” [20] participated in the present study (see Table 1). All participants were recruited from the university sport science department student population. This study was registered retrospectively on ClinicalTrials.gov (registration number: NCT07466628), approved on 17 November 2024. At the time of study initiation, the research team did not realize that prospective registration was required for a non-CTIMP, nutrition-based crossover study involving healthy participants and sporting performance outcomes. Upon becoming aware of journal and transparency requirements, the study was registered to ensure public accessibility and accountability. The study protocol, eligibility criteria, intervention conditions, and primary and secondary outcome measures were defined prior to participant recruitment and were not modified following study commencement. No changes were made to the study design or outcomes after registration.

The inclusion criteria were healthy adults (18–30 years) who were injury-free, with previous weight/strength training experience (≥2 years). The project also opened participation to females, but no females volunteered. Participants habitually retired to bed between 22:00 and 23:30 h and rose at 06:00–07:30 h and for the study purposes agreed to retire at 22:30 and rise at 06:30 h. None of the participants were receiving any pharmacological treatment (including non-steroidal anti-inflammatory drugs, NSAIDs) throughout the study period. Habitual caffeine consumption was assessed using the caffeine consumption questionnaire (CCQ), and those with >150 mg per day were excluded [22]. Further, all participants expressed no preference to training regarding time of day by a weekly self-reported 2-week training diary. Recruiting participants with this specific type of exercise history meant that the known neuromuscular facilitative responses, which are typically associated with acute increases in muscular strength amongst untrained individuals due to neural adaptations and responses, were reduced [23]. The exclusion criteria included depressed mood (from the Beck depression inventory), poor sleep quality (a Pittsburgh sleep quality index global score >5 [24]), recent shiftwork or travel across multiple time-zones and ‘extreme’ chronotype (assessed via the Composite Morningness Questionnaire [25]) and risk factors and/or symptoms of cardiovascular disease. Through interviews, it was established that the volunteers had minimal knowledge of the effects of time of day or time since sleep on human performance. Verbal explanation of the experimental procedure was provided to everyone; this included the aims of the study, the possible risks associated with participation and the experimental procedures to be utilized. Any questions were answered. Individuals then provided written, informed consent before participating in the study. The experimental procedures were approved by the Human Ethics Committee at Liverpool John Moores University (ETHICS CODE: 08/SPS/030). The study was conducted in accordance with the ethical standards of the journal and complied with the principles of the Declaration of Helsinki.

### 2.2. Research Design

**Figure 1:**
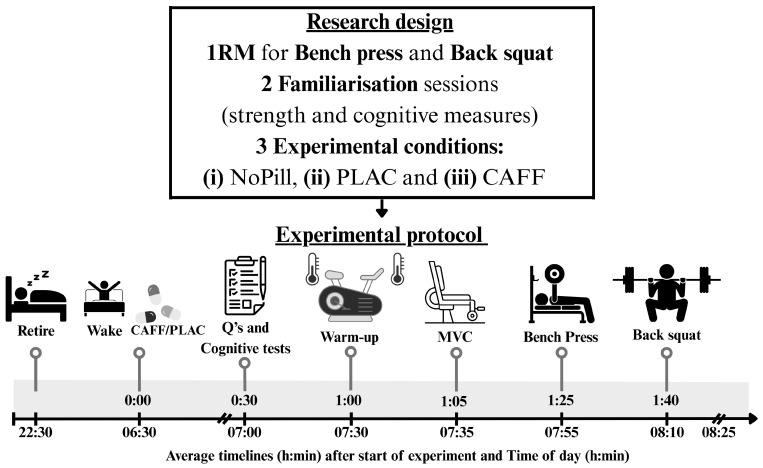
Schematic representation of the research design and experimental protocol undertaken by participants in the investigation. Pills were taken upon wakening; first timeline starts from arrival at the laboratory (Lab). The thermometer represents T_rec_ and T_sk_ measurements.

All participants were required to visit the physiological laboratory (Liverpool, UK) on seven occasions (dry temperature of 19–21 °C, 29–41% humidity, barometric pressure of 962–1010 mmHg and ambient light of 750 lux). Participants completed (i) one maximum repetition (1RM) for bench press and back squat, (ii) two familiarization sessions of cognitive and strength performance tests one week prior to the experimental protocol—during familiarizations, participants completed a 7-day food diary that was analysed using Nutritics^®^ analysis software (2026, Education Edition, version 6.15, Ireland by an SENr-registered sports and exercise nutritionist—and (iii) three experimental sessions, all at least 72 h apart as a standardized recovery period. The experimental part comprised three experimental trials, (a) control (NoPill), (b) caffeine (300 mg, CAFF) and (c) placebo (maltodextrin, PLAC), between 07:30 and 08:30 h (Figure 1). Participants retired the previous night around 22:30 h, woke up at 06:30 h and arrived at the laboratory at 07:00 h in a fasted state (Figure 1). For the CAFF and PLAC conditions, pills were provided in a plastic bottle with instructions to consume with water at 06:30 h prior to arrival at the laboratory, with confirmation upon arrival. For the NoPill control, nothing was provided. Participants were instructed to avoid training or intense physical activity 48 h prior to any of the visits; otherwise, participants maintained their normal daily routines. In total the three caffeine capsules contained 300 mg of caffeine anhydrous, and the remaining space was occupied by maltodextrin (Applied Nutrition Ltd., Knowsley, UK); the placebo capsules were made in the department and contained maltodextrin (~2.4 g, Sport supplements Ltd. t/a BulkTM, Colchester, UK). Researchers and participants were blinded to the supplement schedule, and both caffeine and the placebo were lightly dusted with maltodextrin to create a similar taste; both had similar weight (0.8 g/capsule) and were 00 size. The order for treatment was revealed at the end of the project by a researcher responsible for anonymization and randomization (BE).

All trials were performed with only one participant at a time, but with a staggered start so one participant came in at 06:45, the next at 07:00 h and the last for that morning’s session at 07:15 h. This scheduling for participants was kept consistent for all sessions. All participants lived no more than 10 min away from the university laboratories. Participants were equally allocated, based on their 1RM for bench press and back squat into three groups (A, B and C), by listing participants’ times from the second familiarization in order from the strongest to the weakest and assigning the letters A, B and C in order. The incidence of the learning effect [16] was minimized by assigning the experimental conditions in counterbalanced order. The present study was carried out between the months of November and February (UK autumn to winter), with morning sunlight exposure limited to <80 Lux before arriving at the laboratory. The sunrise range for the duration of the study was 07:12 to 07:03 h.

### 2.3. Protocol and Measurements: Familiarization Session

The participants completed two familiarization sessions of all the strength, cognitive and questionnaire measures before being considered ready to participate in the study. A coefficient of variation value less than or equal to 10% for variables between the first and second measures was used as criteria for familiarization in alignment with the recommendations given by Atkinson and Nevill [26]. Familiarization sessions took place at 12:00 h over a three-week period and finished one week before the study commenced to minimize learning effects. Participants arrived 0.5 h before the start of the test and rested in a seated position, to minimize the influence of prior muscle activity. Participants completed the familiarization in the following order: (i) cognitive battery of tests, (ii) isometric maximal voluntary contraction (MVC) with and without percutaneous electrical stimulation, and (iii) back squat and bench press.

*(i)*  *Cognitive battery of tests*

Trail-making test (TMT; parts A and B): Both parts of the TMT consist of 25 circles distributed over a sheet of A4 paper. In part A the circles are numbered 1–25, and the participant is instructed to draw lines to connect the numbers in ascending order. In part B, the circles include both numbers (1–13) and letters (A–L), and the participant is instructed to draw lines to connect the circles in an ascending pattern but with the added task of alternating between numbers and letters (i.e., 1-A-2-B-3-C, etc.). In both parts the participant is instructed to connect to the circles as fast as possible, without lifting the pencil from the paper. If an error is made, this is pointed out immediately and the participant is allowed to correct it. During the test, time to completion is measured, with a higher time indicative of the greater impairment [27].

Rey auditory verbal learning test: The RAVLT is a neuropsychological assessment designed to evaluate verbal memory in patients aged 16 years and older. The RAVLT can be used to evaluate the nature and severity of memory dysfunction and to track changes in memory function over time [28]. The test is designed as a list-learning paradigm in which the volunteer hears a list of 15 nouns and is asked to recall as many words from the list as possible. The number that are correct and the number that were given by the participant but are not on the list (intrusions) are noted. This process is repeated 4 more times. The process is repeated but with a second “interference” list (List B), which is presented in the same manner, and the participant is asked to recall as many words as possible from List B, and the score is recorded. After the interference trial, the participant is immediately asked to recall the words from List A, which they heard five times previously, and the number of correct words and the number of intrusions are recorded. The RAVTL total number, number of distractions and retention are recorded and analysed.

Stroop word–colour interference test [29,30]: The participants were asked to read out their responses to words or colours for 45 s as quickly as possible and to leave no errors uncorrected. This was filmed, and the number of errors and total amount completed were recorded and analysed. The first sheet had text (red, blue, yellow, black and green) in black ink (naming word test, W). The second sheet had blocks of colour corresponding to the text on the first sheet (naming colour test, C). With the third sheet, the participants had to read out the word (which was coloured differently to the word, e.g., the word was yellow and the colour red, referred to as the naming colour of word test, CW), and for the fourth sheet, the participants had to read out the colour (which was wrongly named, e.g., the colour was yellow but the word was red, referred to as the naming of word not colour test, WC). In this fourth sheet, the words were printed in the reverse order to the third sheet. The raw data was analysed for the number of errors (to give an indication of pacing/speed accuracy in the 45 s) and interference scores (an indicator of the efficiency of the inhibitory function), where C represents the correct answers produced in 45 s in naming colours, and CW corresponds to the correct answers achieved in 45 s in the interference condition [31]. This was also performed for the naming of word not colour test.

I = [(C − CW)/(C + CW)] × 100(1)

*(ii)*  *Isometric maximal voluntary contraction*

Participants performed isometric MVCs of the quadriceps muscles (3 s duration), both with and without twitch interpolation percutaneous stimulation (Digimeter, DS7, Hertfordshire, UK). During the initial session, participants practised performing MVCs without twitch interpolation, to become accustomed to the practice of achieving and maintaining voluntary force for the time required. This session was also used to obtain maximal current tolerance and establish the supra-maximal current amplitude for superimposition during an MVC. With the subject at rest, the amperage of a 240 V square-wave pulse (100 μs, 1 Hz) was progressively increased until the point at which further increases in intensity caused no further increase in resting twitch force reached [32,33].

#### Twitch Interpolation

The quadriceps were electrically stimulated using two moistened surface electrodes (Chattanooga, TN, USA, 5 × 12 cm), which were positioned proximally over the vastus lateralis and distally over the vastus medialis. The skin was prepared prior to placement of each electrode by shaving and light abrasion of the skin, followed by cleaning with an isopropyl alcohol swab. A permanent marking pen was used to mark and identify the position of each electrode to minimize electrode placement variability from session to session [16,34]. Two impulses were delivered before and after the contractions; the other two impulses were administered during the contraction period and tested the peak value of the MVC. The peak forces of the pre- and post-contraction twitches were then averaged, allowing comparison of resting twitch amplitudes in both an unpotentiated and potentiated condition, respectively [35]. The amplitude of supra-maximal superimposed current was identified for each subject in familiarization sessions and corresponded to 10% above the level required to evoke a resting muscle twitch of maximal amplitude [36].

Data was acquired for nine seconds and analysed with a commercially designed software program (AcqKnowledge III, version 5.0.2, Biopac Systems, Goleta, CA, USA). The calculation of voluntary activation was conducted according to an interpolated twitch ratio (as recommended by Merton, 1954 [37]), whereby the size of the interpolated twitch is expressed as a ratio of the amplitude elicited by the same stimulus delivered to a relaxed potentiated muscle. The average force during the 100 ms^−1^ period before the application of each stimulus during the contraction was recorded, and subsequently the maximal force during the 100 ms^−1^ period after each stimulus had been applied was also recorded. The 100 ms^−1^ mean pre-stimulus force (taken as MVC force) and the resulting maximal post-stimulus force were used to calculate the size of the interpolated twitch, by subtraction of the mean pre-stimulus force from the maximal post-stimulus force.

Voluntary Activation = [1 − (Size of interpolated twitch/Size of resting twitch)] × 100(2)

During familiarization sessions, participants alternated between performing MVCs with and without twitch interpolation, so that approximately three trials of each were performed within each session. This approach was suggested by Morton et al. [33], who reported that many participants performed weaker contractions when they were expecting stimulation (attributed to the potential apprehension due to the prospect of receiving noxious stimuli) compared to when they were not expecting stimulation. Standardized strong verbal encouragement during each familiarization session/trial and real-time visual feedback of their performance via the computer display on a large screen placed in front of the participants were provided.

*(iii)*  *Back squat and bench press*

Participants were familiarized with back squat and bench press three times prior to commencement of the testing cycle. Each participant was asked to perform the back squat with incremental loads (40%, 60% and 80% 1RM) for one repetition at each load, and 5 min rest was allowed between each effort. Likewise for bench press, each participant performed one repetition against each incremental load (40%, 60% and 80% 1RM), and again 5 min rest was given between each working effort. This was done so that the upper loads required for the experimental trials were known to be comfortably within each of the participants’ physical capabilities, and as such there was minimal likelihood of them failing to perform the required efforts for data collection. Each of the participants performed the two exercises in same order: bench press and then back squat, performing three lifts at each exercise against progressively increasing loads (40%, 60% and 80% 1RM). The individual’s own body mass was factored into the back squat exercise, as this is a whole-body movement, but not into the bench press. The MuscleLab force–velocity linear encoder (Muscle Lab, Ergotest version 4010, Stathelle, Norway) was attached to an Eleiko Olympic bar (20 kg), which was set upon rests on a standard squat rack; safety arms were set so that the participant achieved ≤90° knee flexion position (settings measured and recorded during the familiarization process). From this position, the participant was instructed to drive the bar upwards as forcefully as possible; the value recorded during the test was for the concentric phase of the action only. The MuscleLab system encodes the force, the displacement of the bar, and the velocity produced for each individual lift, so that following measurements of muscle performance can be measured: average velocity (AV), average power (AP, average force × average velocity), peak velocity (PV, highest value of velocity), time to peak velocity (tPV, time to reach the highest value of velocity), mean propulsive velocity (MPV, average velocity during the propulsive phase of the concentric movement), rate of development of velocity (RDV, average acceleration during the propulsive phase of the concentric movement) and displacement (D, linear displacement of the bar during the movement). This process was repeated three times, with 5 min rest allowed between each individual lift, and against the progressive workloads as described above. For the bench press, the participant was instructed to lower the bar to their chest and, again, the instruction given was to push against the bar as forcefully as possible. This was repeated three times against the workloads described above, with 5 min rest between each push.

The highest of the three AP outputs (and associated AV, PV, MPV, RDV, D and tPV values) was used for analysis for each mass on the bar for both bench press and back squat. To reduce the likelihood of injury, two people were positioned either side of the participants as they performed their lifts, to intervene at any time if needed. In addition, there was safety support in place on every occasion.

### 2.4. Experimental Protocol and Measurements

The experimental sessions took place a week after the last familiarization, with 72 h recovery between the three experimental conditions (i.e., CAFF, PLAC, and NoPill). Upon arrival at the laboratory, participants were asked to insert a soft flexible rectal probe (mini-thermistor, Grant Instruments, Shepreth, UK) approximately 10 cm beyond the external anal sphincter (Figure 1). Participants then rested for 30 min in seated position to assess resting rectal temperature (T_rec_). Skin temperature (T_sk_) was assessed simultaneously by skin thermistors (Grant Instruments, Squirrel 2010 series, Shepreth, UK), which were placed at four locations on the left side of the body (chest [ch], forearm [f], thigh [th] and calf [ca]). The average of the last 5 min of the 30 min resting period was recorded for resting T_rec_ and T_sk_ temperatures. Mean T_sk_ was calculated as follows [38]:

T_sk_ = (0.34 × T_ch_) + (0.33 × Tth) + (0.18 × T_ca_) + (0.15 × T_f_) (3)

The mean body temperature (T_mb_) was calculated using [39]:

T_mb_ = (0.64 × T_r_) + (0.36 × T_sk_). (4)

Participants completed the profile of mood states questionnaire (POMS; Version 32 [40]), their subjective rating of sleep (using sleep questions from the Liverpool Jet lag Questionnaire [41]), effort (0–10, 0 = no effort and 10 = maximal effort) and rating of perceived exertion (RPE) [42]. After the initial rest measurements, participants warmed up on a cycle ergometer (Corival cpet, Lode, Groningen, The Netherlands) for 5 min at 150 W [43]. Post-warm-up, participants removed the rectal probes in private, and the skin thermistors were taken off, after which MVC and bench press and back squat exercises were then undertaken.

### 2.5. Statistical Analysis

The main research variables in the present study were peak force in the MVC and PV in the bench press and back squat. For MVC peak force, sixteen participants were estimated to provide 80% statistical power (*p* < 0.05), with a diurnal variation effect size (ES) of 0.67 [3] for a one-tailed *t*-test. For PV for bench press, nine participants were estimated to provide 80% statistical power (*p* < 0.05), with a diurnal variation effect size (ES) of 0.92 [3] for a one-tailed *t*-test. For PV for back squat, nine participants were estimated to provide 80% statistical power (*p* < 0.05), with a diurnal variation effect size (ES) of 0.91 [3] for a one-tailed *t*-test. We recruited 18 participants to allow for dropout, with *n* = 4 unable to complete the full protocol. The sample size estimation was performed using software G*Power 3.1 [44]. The data was analysed using the Statistical Package for Social Sciences version 30 (SPSS, Chicago, IL, USA). All data was checked for normality using the Shapiro–Wilk test. General linear models were used to analyse all measurements collected; the significance of interactions and main effects were evaluated by ANOVA. Sphericity violations were corrected by Greenhouse–Geisser (ε < 0.75) or Huynh–Feldt correction (ε > 0.75) and main effects by Bonferroni pairwise comparisons. Pearson correlations were performed to explore individualized responses to CAFF vs. other conditions (relative to body mass) and % change in MVC performance. All data is presented as means ± standard deviation (SD), unless otherwise stated. Significance was set at *p* ≤ 0.05. *p* Values between 0.05 and 0.10 were considered to indicate a statistical trend [45]. Effect sizes are referred to as partial eta squared values (η^2^_p_), with values of 0.01, 0.06 and 0.14 corresponding to a small, medium and large effect, respectively [46]. Ninety-five-percent confidence intervals (95% CI) and mean differences between pairwise comparisons are reported where suitable.

## 3. Results

### 3.1. Rest and Post-Warm-Up Temperatures

There were no significant main effects of experimental conditions for T_rec_, T_sk_ and T_mb_ at rest (*p* > 0.05, Table 2). There was a main effect for “rest to post”, where T_rec_ increased and T_sk_ and T_mb_ values decreased from rest to post-warm-up, respectively (mean difference = ↑0.13 °C CI = 0.04 to 0.23 °C, *p* = 0.012; mean difference = ↓0.97 °C CI = 0.73 to 1.20 °C, *p* < 0.001 and mean difference = ↓0.28 °C CI = 0.20 to 0.37 °C, *p* < 0.001). There were no significant interactions.

### 3.2. MVC Force and % Activation

**Figure 2:**
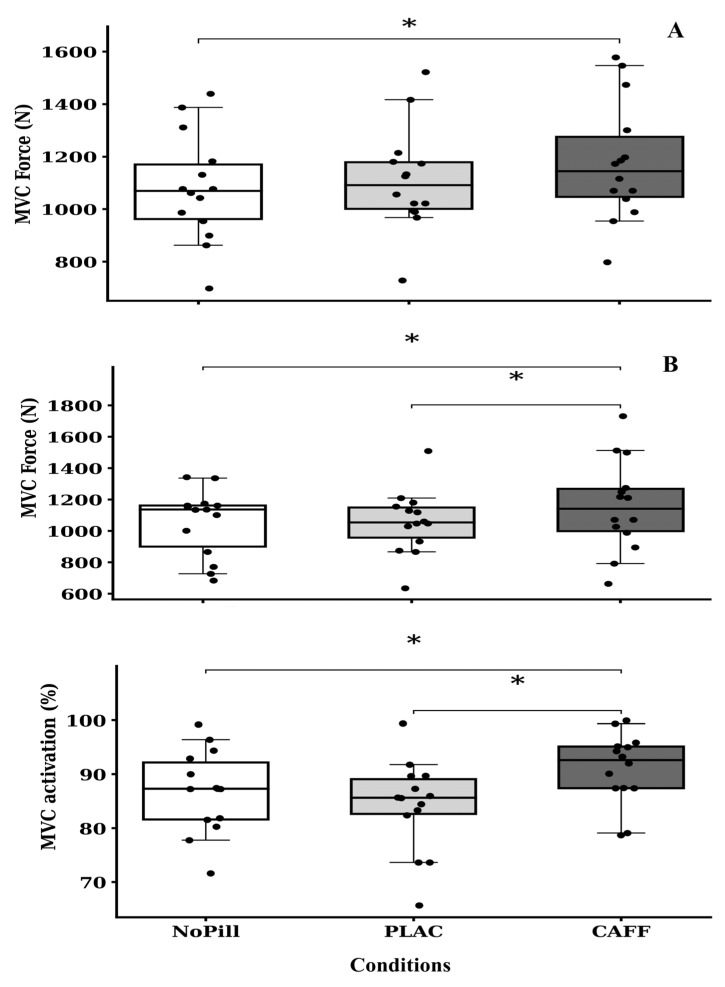
Individual and median (±95% CI) values of isometric maximum force production without stimulation (**A**) and with stimulation (**B**) and muscle % activation for the three experimental conditions (NoPill, PLAC, and CAFF). * denotes main effect for condition as shown by Bonferroni pairwise comparisons (*p* < 0.05).

There was a significant main effect of condition for isometric MVC maximum force, with (*p* = 0.008) and without stimulation (*p* = 0.001). With stimulation, post hoc comparison revealed CAFF condition peak force was higher than the NoPill values (mean difference = 103.9 N, CI = 1.9 to 205.9 N, *p* = 0.046) and PLAC conditions (mean difference = 100.9 N, CI = 1.2 to 202.9 N, *p* = 0.050). Without stimulation, CAFF condition force was higher than the PLAC (mean difference = 98.4 N, CI = 54.6 to 142.3 N, *p* < 0.001) but not significantly different from the NoPill condition (mean difference = 67.5 N, *p* = 0.067; see Figure 2 and Table 2). There was a main effect of condition for muscle % activation where, in the stimulated MVC, the level of activation was higher in the CAFF condition than the NoPill or PLAC conditions (mean difference = 4.3%, CI = 0.1 to 8.4%, *p* = 0.042 and 6.9%, CI = 2.4 to 11.3%, *p* = 0.003). In conditions without percutaneous electrical stimulation, general RPE values were lower in the CAFF than NoPill and PLAC conditions (16.9 vs. 17.8 and 17.7 AU, *p* =0.040; Table 2). No other variables showed significance. The presence of a greater increase in peak force in isokinetic maximum voluntary contraction in the CAFF condition than either NoPill or PLAC values addresses a principal aim of this research project.

### 3.3. Bench Press

**Figure 3:**
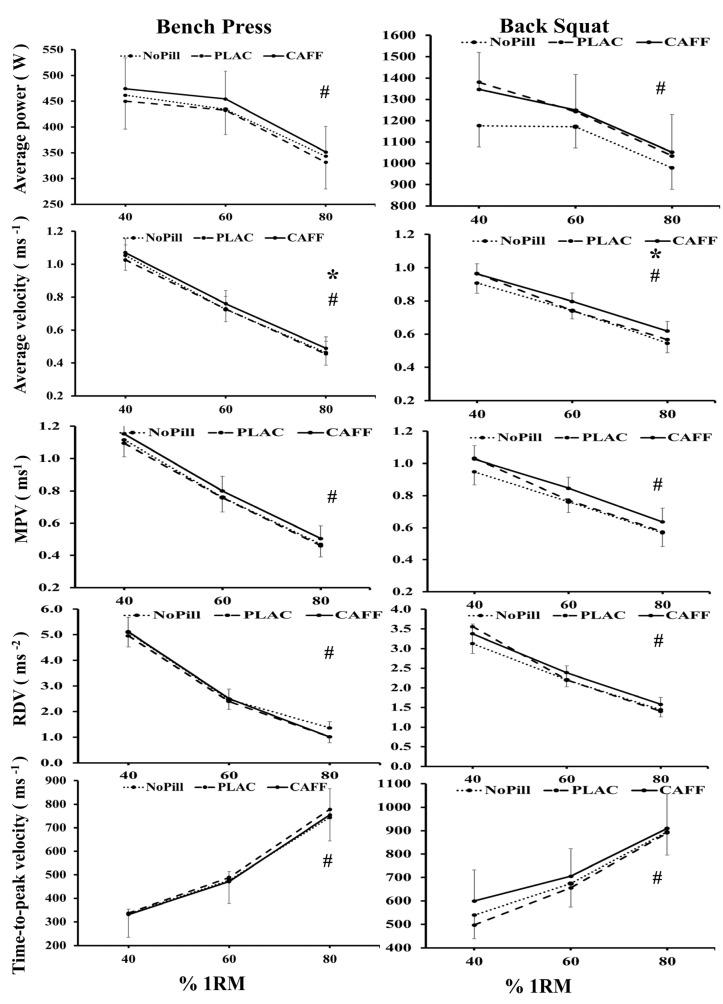
Mean and 95% CI values of each performance variable for morning (07:30 h) bench press and back squat at 40%, 60% and 80% 1RM loads for the three experimental conditions (NoPill, PLAC, and CAFF). * denotes main effect for condition; ^#^ denotes main effect for load as shown by Bonferroni pairwise comparisons (*p* < 0.05). RDV and MPV denote rate of development of velocity and mean propulsive velocity.

There was a significant main effect of condition for AV and MPV, where both indices were higher in the CAFF than NoPill or PLAC condition (mean difference = 0.037 ms^−1^, CI = 0.005 to 0.068 ms^−1^ *p* = 0.031 and 0.48 ms^−1^, CI= 0.06 to 0.103 ms^−1^; *p* = 0.031; see Table 2). There was a significant main effect of ‘load’ for all bench press variables measured (Table 2, Figure 3). For AP, AV, D, MPV, RDV and PV, values were highest at 40% of 1RM and lowest at 80% 1RM (mean difference = 119.77 W, 0.58 ms^−1^, 4.98 cm, 0.64 ms^−1^, 3.92 ms^−2^, 0.93 ms^−1^, respectively), whereas tPV was significantly lower at 40% 1RM than at 80% 1RM (mean difference = 424.6 ms^−1^). As expected, there was a corresponding significant main effect of ‘load’ on subjective effort and RPE values (*p* < 0.05). Lower subjective values were observed at 40% 1RM load, whereas 80% 1RM elicited the highest values [RPE general (6–20): 5.7; RPE effort (0–10): 4.0; exertion in task: 3.5 (1–10)]. There was no significant interaction of ‘condition and load’ for any variables, such that values across all conditions at both time points for the three loads rose or fell in the same manner (see Figure 3).

### 3.4. Back Squat

There was a significant main effect of condition for AV, MPV and D, where both indices were higher in the CAFF than NoPill or PLAC condition (mean difference = 0.03 ms^−1^, 0.08 ms^−1^ and 2.6 cm; *p* > 0.05; see Table 2) or PLAC values (mean difference = 0.06 ms^−1^, 0.04 ms^−1^ and 2.7 cm; *p* > 0.05; see Table 2). There was a significant main effect of ’load’ on all back squat variables *(p* = 0.173; see Table 2). As expected, AP, AV, D, MPV, RDV and PV were highest at 40% of 1RM load and lowest at 80% of 1RM load (mean difference = 279.3 W, 0.37 ms^−1^, 8.8 cm, 0.41 ms^−1^, 1.88 ms^−2^, 0.53 ms^−1^, respectively), whereas tPV was significantly lower at 40% 1RM and highest at 80% 1RM (353.7 ms^−1^). There was a corresponding significant main effect of ‘load’ on subjective effort and RPE values (*p* < 0.05). At 40% 1RM, there were lower subjective values, whereas 80% of 1RM elicited the highest values [RPE general (6–20): 5.3; RPE (0–10): 3.8; exertion in task: 3.0 (1–10)]. There was a significant interaction of ‘condition × load’ for MPV and RDV variables, where CAFF and PLAC condition RDV values are greater than NoPill at 40%, but from 60 to 80% 1RM only CAFF values are higher than NoPill values (Figure 3). The presence of a greater increase in AV and MPV in bench press and back squat values in the CAFF condition than either NoPill or PLAC addresses a principal aim of this research project.

### 3.5. Cognitive Function Tests

There was a main effect for RAVLT words total number (*p* = 0.040), where the total number correct was higher in the CAFF condition than NoPill (mean difference = 5.2, CI = 0.65 ± 11.08, *p* = 0.040). Post hoc comparisons revealed no significant differences between CAFF and PLAC conditions (*p* > 0.05, Table 3). No other variables showed a significant effect for condition.

### 3.6. Resting Profile of Mood States, Caffeine Withdrawal and Sleep Questionnaires

There was no significant effect of condition for all mood profiles (anger, calm, confusion, depression, fatigue, and happiness), nor for caffeine withdrawal symptoms or sleep questionnaires (see Table 4).

### 3.7. Correlations Between Dose of Caffeine Ingested and Changes in Peak Force from Placebo and No Pill

**Figure 4:**
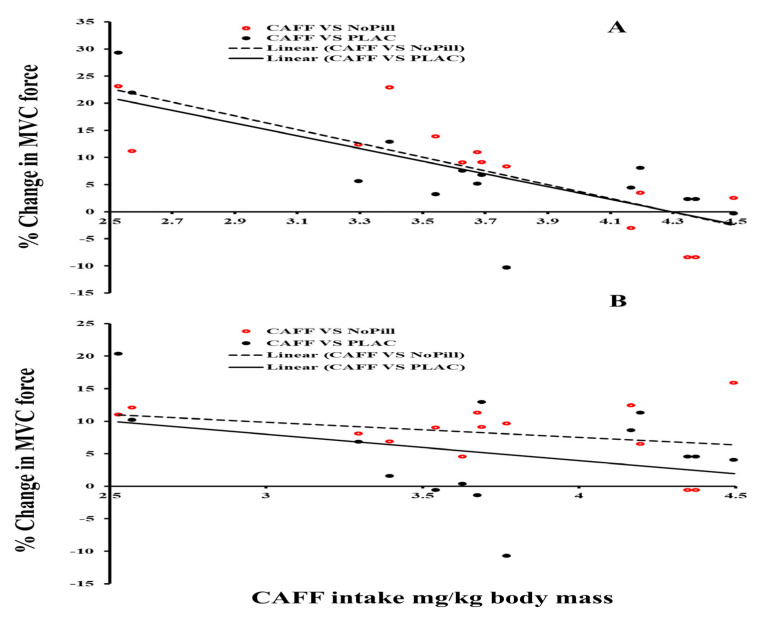
Relationship between % change in morning isometric maximum voluntary contraction force (MVC) from a placebo (solid line) and from no-pill control (dashed line) expressed in mg/kg of caffeine ingested (CAFF intake/kg body mass). MVC with (**A**) and without percutaneous electrical stimulation (**B**).

Pearson correlation showed a significant negative linear relationship between individual dose of caffeine % change in MVC vs. NoPill when electrical stimulation is superimposed (*n* = 14, r =−0.661, *p* = 0.01, Figure 4), where lower dose per kg of body weight results in a greater change in force production. Lastly, there was no significant correlation between dose and PLAC vs. NoPill with stimulation (*p* = 0.325 and *p* = 0.556).

## 4. Discussion

The main aim of this study was to assess the effect of 300 mg of caffeine (~3.6 mg·kg^−1^ body mass) ingested in the morning on subsequent (~1–1.5 h later) strength and power output and cognitive performance (including tasks of attention, memory, and executive function). In recreationally active males, who were deemed low habitual caffeine users (by consumption questionnaire [22] and dietary intake analysis 110 or 37.3 mg.day^−1^; Table 1), we found that acute caffeine supplementation improved peak isometric force compared to NoPill and PLAC conditions (with stimulation, Δ+8.7 and +9.0% with a large effect size of η^2^_p_ = 0.31). Further, caffeine improved peak isometric force without stimulation compared to the NoPill condition (Δ+8.3% with a large effect size of η^2^_p_ = 0.42). These values are higher than those reported in a meta-analysis that did not consider time of day, whereby caffeine ingestion improved MVC strength by 4% compared to a placebo [47]. Average and mean propulsive velocity in bench press and back squat were also higher in CAFF compared to the NoPill and PLAC conditions, with large effect sizes, whatever the modality of testing and the variable (η^2^_p_ between 0.23 and 0.32), and larger variation for back squat modality (from Δ+3.3 to +4.6% to +7.7 to +9.2%). These findings agree with our first hypothesis in individuals who followed a standardized protocol that incorporated some of the main concerns in the literature regarding chronobiological design [15], but unlike other studies, we utilized a study design that incorporated a NoPill group in conjunction with the CAFF and PLAC conditions. This approach ensured that any placebo effect is accounted for and that the true potential effect of the supplement can be established [6,48]. To the best of our knowledge, the current study is the first to investigate the effectiveness of acute caffeine consumption on morning peak isometric force. The increase in peak force with stimulation in the CAFF condition was associated with higher levels of voluntary muscle activation than the NoPill or PLAC conditions (+7.6 and +4.7%). Furthermore, the increase in peak force with no stimulation in the CAFF condition was associated with a lower general rating of perceived exertion than in both the NoPill and PLAC conditions (Δ+5.1 and +4.5%, *p* = 0.040; Table 2). These results are in agreement with the mechanism of action of caffeine, to blockade adenosine receptors, increase neural activation, and enhance calcium release in muscles. This reduces perception of effort and fatigue, making it easier to exert maximal effort [11,12,13,49]. Both variables in bench press and back squat were greater in CAFF than NoPill and PLAC conditions. Larger increases were observed between CAFF and NoPill (+8% to 10%), which may denote a slight placebo effect. Unlike other studies, the present work used a study design that incorporated a NoPill group in conjunction with the CAFF and PLAC conditions. This approach ensured that any placebo effect is accounted for and that the true potential effect of the supplement can be established [6,48]. Average power during bench press tended to show higher value in CAFF condition, but the differences did not reach the significant level (*p* = 0.062, η^2^_p_ = 0.20, large effect size). Taken together, these results agree with others that showed improvements in morning values of dynamic strength and power output of upper and lower muscle groups after caffeine ingestion [9,10]. In these studies, resistance-trained participants consumed similar caffeine doses at an interval between ingestion and performance assessment equivalent to the current study (3 mg·kg^−1^ body mass, ingested 60 min before the experimental session). However, measurements were undertaken at later times of day (09:00–10:00 h) and did not include a no-pill control intervention [9,10].

A significant main effect for “load” was present for all bench press and back squat output variables (AP, D, AV, PV, tPV, MPV and RDV; see Table 2). From 40 to 80% 1RM, where there was greater load against the movement, tPV increased as expected, due to the longer duration for movement. Other variables such as PV, AV, AP, MPV and RDV (Figure 2) were highest when the load on the bar was lowest (40% 1RM). Additionally, ratings of subjective effort and RPE values increased in line with the increasing load on the bar. These findings are consistent with the fundamental force–velocity properties of skeletal muscle and have been demonstrated by us and others previously during submaximal loads using MuscleLab linear encoders and force platforms during complex movements [3,50]. Others have reported improved muscular strength/power variables at moderate-to-high loads (75–90% 1RM) and endurance performance (65% 1RM) in the back squat while counteracting morning declines at light load (25% 1RM) for both back squat and bench press (Table 5) [10]. Similarly, we found that during the back squat, which has a large cognitive component that is associated with ‘high-skilled’ movements, there was a significant interaction of ‘condition and load’ for MPV and RDV variables, where CAFF and PLAC condition RDV values are greater than NoPill at 40%, but from 60 to 80% 1RM, only CAFF values are higher than NoPill values (Figure 1). Cognitive performance has been linked with mood and arousal [51], where mood state is a major factor that influences motivation and thus weightlifting performance [2,52]. We observed no effect of CAFF on mood in the present study, unlike others, who found a greater increase in anxiety and vigour after the CAFF than PLAC [11,53].

A second aim of the current study was to investigate the effect of caffeine ingestion on morning cognitive performance (including tasks of attention, memory and executive function). There is no consensus in the literature regarding the effect of acute caffeine consumption on cognitive performance [54,55] due to different doses of caffeine and choices of cognitive tests. In the present study, the only variable of cognitive function that showed a CAFF ingestion effect was the total number in Rey’s auditory verbal learning test [28]. Differences were found between CAFF and NoPill, with higher values in the CAFF condition, but not between CAFF and PLAC values, which indicates a possible placebo effect. To identify nocebo effect (i.e., bias due to thinking you were on the PLAC condition negatively influencing your performance [48]), our protocol required participants to indicate which condition they thought they were on, both when they started the cognitive tests and after the physical strength tests, with participants unable to identify which condition they had undertaken. Therefore, it could be assumed that the placebo effect acted via the maltodextrin contained in the capsules.

Although there is a large body of research into the use of caffeine and gross muscular maximal or submaximal performance [48], mood [56] or cognitive function [57], investigations into morning performance in strength are scarce, and even more so regarding the effects on cognitive function (Table 4). Considering only research that investigated the effects of acute caffeine ingestion in the morning on strength and/or cognitive function in a thermoneutral laboratory (~19–21 °C) in adult males, our findings of greater strength with caffeine vs. PLAC agree with 5/11 of the previous research [8,9,10,11,53,58,59,60,61]. However, it is challenging to compare our findings directly with others as there are discrepancies in approaches between studies. We employed a standardized approach as called for in human chronobiology research [17,62,63], and specifically in chronobiology and strength investigations [3]. To do this, we collected the advice of several sources to reduce bias (of measurement and reported result) by (i) participant considerations, (ii) methodological and equipment considerations, and (iii) environment considerations (Table 4). We applied this advice in the design of the current study to reduce the signal-to-noise ratio, to allow the effects of caffeine on morning performance, should they exist, to be identified. For some recommendations, we have been pragmatic, so we did not adopt times of day of measurement such as 04:00–05:00 h (body temperature minimum) that would cause sleep loss. If caffeine is known to restore performance under conditions of sleep disturbances or restriction, it is important to assess its effectiveness on restoring morning performance under habitual sleep conditions without explicit sleep loss or circadian disruption [64].

In the 11 studies from the literature (Table 5), the sample sizes ranged from 12 to 48, with only 3/11 papers being a priori power tests. Training status is either quantified or just stated; a method of categorizing fitness such as McKay et al. [20] would help comparison of populations between studies. Further, only 5/11 papers recorded metrics of years of strength training and fitness such as 1RM bench press or back squat or McKay’s fitness data, again enabling population comparisons between studies. The chronotype scores of the population were recorded in 3/11 studies, but habitual sleep time was only reported in two studies; recruiting a participant whose sleep habits are vastly different to those imposed by the protocol could cause detriments to the outcome measures. In more recent work, it has been highlighted that the inclusion/exclusion criteria to reduce confounding variables in studies should also include “not” receiving pharmacological treatment (including NSAIDs), a habitual caffeine “low” consumption, hence <150 mg per day assessed by the caffeine consumption questionnaire, as well as drug and alcohol considerations [62]. The strength measures ranged from handgrip to back squat to bench press. The cognitive tests were “academic type” and reaction time (modified Flanker Task). Allocation to groups was generally randomized, counterbalanced, and double-blinded, with the time between sessions being 24 h to 5 days to allow recovery. In no studies was there a ‘NoPill’ condition. Diet restrictions were considered in 6/11 studies following the general advice of Drust et al. [2]; therefore, we similarly chose to follow this where participants recorded the type, amount and timing of the food they ate for the 24 h period before the day of the first session and were asked to replicate this diet for the days before the second experimental condition. Individuals came in having fasted for the morning sessions. Again, we took a pragmatic approach to diet as abstinence of food in the morning has shown no effect on performance compared to having breakfast [65]; however, a more stringent control of diet the day before the experimental day where the effects of the timing (relative to the session), amount (energy yield), and composition (carbohydrates, protein, and fat) of food consumed during a study may be a confounding factor in performance [17]. The measurement site for core body temperature in the research was generally intra-aural (3/11 studies); this site is subject to masking effects specifically in the morning and can confound any daily variation [41]. Five studies did not report core temperature but relied on this as the main mechanism to describe diurnal variation in time-trial performance in their discussion. Time-of-day scheduling was generally from 06:30 to 11:00 h with laboratory temperature, humidity and barometric pressure only recorded in two studies. The error in measurement of strength parameters chosen for the strength measures was not reported. Time of year—hence photoperiods or exposure to daylight in the morning, coming into the laboratory—was poorly reported (0/11 studies) [66].

Lastly, familiarization was either one session completed (4/11) or several (6/11). The effect of familiarization of the strength and cognitive performance measures and the number of sessions/practice trials needed to make subsequent learning effects negligible is unclear even if the order of administration of the tests is counterbalanced using a cross over-repeated measures design. If you measure it, you can quantify the random error and systematic bias of your population by following your specific protocol.

One of the proposed mechanisms for caffeine improving physical performance is by increasing metabolism. Although resting metabolic rate was not assessed in the current study, no significant increase in T_rec_ values was found in the CAFF condition at rest compared to the other conditions (Table 2). We did, however, find an increase with warm-up as expected and a decrease in T_sk_ and T_mb_ to help mitigate heat production at values we have previously observed for 5 min at 150 W in the morning [67,68]. A lack of increase in core body temperature with caffeine intake has been shown by other authors [69], although they used less accurate measures of core body temperature (oral or intra-aural). We did not measure the pharmacokinetics of caffeine in the blood as we do not know when the drug peaked; rather, we presumed 1 h after ingestion for temperature and cognitive performance and 1.5 h for strength measures would be appropriate to elicit a measurable effect [48]. This is despite the fact that there is evidence that humans are in a heat gain mode in the morning, and hence their sensitivity to thermoregulatory changes; in particular, heat loss is lower in the morning [67]. This would result in more heat gain and hypothetically increase the likelihood of a temperature rise, especially in habitually low caffeine users even with moderate intake of caffeine.

To assess individual differences in relation to a dose response for supplementing CAFF, we conducted correlations between CAFF dose and accounted for body mass (kg) and change in performance (isokinetic MVC maximum force output with and without stimulation). Pearson correlation showed a significant negative linear relationship between individual dose of CAFF compared to values from the NoPill condition on isometric MVC maximum force output with stimulation, where a lower dose per kg of body weight resulted in greater % change in force production (r = −0.661, *p* = 0.01). This relationship was not significant for CAFF vs. PLAC with stimulation or CAFF, PLAC and NoPill conditions with no stimulation (Figure 4; *p* > 0.05). Nor was there a significant correlation between dose and PLAC vs. NoPill with stimulation (*p* > 0.05). Even though the current literature on this topic is scarce, contrary to the classic dose–response model that higher CAFF intake produces greater strength related benefits, we found the opposite. However, due to the low sample size, this result should be taken cautiously. Further, in our population of low daily caffeine users, other factors such as individual peak in caffeine uptake and sensitivity to caffeine, which has individual [70] and genetic aspects related to caffeine metabolism could have played a role [54,71,72]. This should be explored in each participant to determine the optimal dosing and timing of ingestion to maximize the effect of the supplement on the individual.

**Table 5:** Comparison of the current study and other authors with the checklist for consideration for diurnal variation (DV) studies by Edwards et al. [14]. Only studies whose participants were male, consumed caffeine and used a measure of strength and/or cognitive function and were undertaken in a thermoneutral environment were included in this comparison. Abbreviations: ME = main effect; TOD = time of day; IDS = individuals; NA = not applicable; CT = core temperature. * indicates the authors were contacted for information.

	Participant Considerations			Methodological and Equipment Considerations				Environmental Considerations		Outcome
Author/Variable	Sample size/Sex/Age/A Priori Sample Test	Training Status/1RM/Performance Information	Habitual Sleep Times/Daily Preferences for Exercise/Chronotype	Strength or Cognitive Assessment/Time Between Sessions/TOD of Sessions	Familiarization with Strength or Cognitive Tests/Experimental Conditions/Allocation of IDs to Sessions/Blinded or Double-Blinded	Diet (Timing/Content) on Day of Testing/Caffeine Consumption from Food Intake and Consumption Questionnaire/Caffeine Dose and Timing	Core Temperature Site (CT)/Kit Isometric Dynamometer and Linear Encoder Chosen for the Strength Tests/Cognitive Tests Chosen	Time of Year/Sunrise and Sunset from Start to Study End	Laboratory Dry Temperature/Humidity/BP	ME Caffeine for MVC/ME for Caffeine for BP/ME for Caffeine for BS/ME Caffeine for CT/ME Caffeine for Cognitive Tests
Current study	14/male/18–28 years/a priori = 16 or 10 for one-tailed *t*-test	Resistance trained > 2 years of strength training 2–4 times per week/1RM BP = 92.5 kg, BS = 122.0 kg	23:30–07:30 h/did not have one/chronotype score = 33 ± 6, all intermediate types	MVC with or without stimulation and BP and BS with LE/battery of cognitive tests/72 h/07:00 h	Three sessions/NoPill vs. PLAC vs. CAFF/allocated to groups due to 1RM BP and BS then counterbalanced/condition double-blinded	Fasting before AM/34 and 100 mg.day^−1^/300 mg (~3.6 mg·kg^−1^ body mass), 60 min before cognitive tests and 9 min before strength tests	Rectal/Squirrel 1000 Series, Grant/Biodex/MuscleLab linear encoder/trail maker, Rays auditory recall (AR), Stroop	October to December (UK autumn to winter)/sunrise–set range from start to end of experiment = 07:29 to 08:01 h and 18:01 to 19:50 h	19 °C/35–45%/750–760 mmHg	**SIM MVC:** CAFF vs. PLAC Δ11.7%; CAFF vs. NoPill Δ9.2% *p* < 0.05 **BP:** CAFF vs. PLAC Δ4.7%; CAFF vs. NoPill Δ3.3% *p* < 0.05 **BS:** CAFF vs. NoPill Δ3.4%; CAFF vs. NoPill Δ4.6% *p* < 0.05 CT: NA **AR:** CAFF vs. PLAC Δ11.7% *p* < 0.05
Montalvo-Alonso et al. [10]	13/male/24 ± 5 years/not given	Resistance-trained; training experience 5 ± 3 years/BP 1RM.kg^−1^ = 1.29 ± 0.16; BS 1RM.kg^−1^ = 1.88 ± 0.34	Not given/not given/not given	BP and BS/3–7 days/09:00 h	One session/CAFF vs. PLA/randomized allocation, counterbalanced/triple-blind (participants, researchers, and analysts) with external researcher holding codes	Not given/8.6 ± 6.7 mg·kg.day^−1^/CAFF 3 mg·kg^−1^ in 150 mL of water, ingested 60 min pre-trial	Not given/equipment: Smith machine (Multipower, Technogym) with Chronojump Boscosystem/cognitive tests not applicable	Not given/not given	Not given/not given/not given	**BP25 Vmean:** CAFF vs. PLAC Δ11% *p* = 0.001 **BP25 Wmean:** CAFF vs. PLAC Δ10% *p* = 0.001 **BP65 endurance Vmean:** CAFF vs. PLAC Δ11% *p* = 0.005 **BP65 endurance Wmean** CAFF vs. PLAC Δ12% *p* = 0.003 **BP65 endurance Wpeak:** CAFF vs. PLAC Δ11% *p* = 0.027 **BS25 Vmean:** CAFF vs. PLAC Δ8% *p* = 0.010 **BS25 Wmean:** CAFF vs. PLAC Δ11% *p* = 0.003 **BS75 Vmean:** CAFF vs. PLAC *p* = 0.001, (Δ% not reported) **BS75 Wmean:** CAFF vs. PLAC *p* = 0.004, (Δ% not reported) **BS90 Vmean:** CAFF vs. PLAC *p* = 0.010, (Δ% not reported) **BS90 Wmean:** CAFF vs. PLAC *p* = 0.043, (Δ% not reported) **BS65 endurance Vmean:** CAFF vs. PLAC Δ8.6% *p* = 0.001 **BS65 endurance Wmean:** CAFF vs. PLAC Δ9% *p* = 0.001 CT: NA; cognitive tests: NA
Köse et al. [57]	17 (8 morning and 9 evening type)/male/22.75 ± 1.98 y and 22.22 ± 1.30 y/a priori = 16	Strength training ≥ 3 days/week (past year)/not given	Not given/not given/morning type and evening type	Handgrip, back strength, cognitive test (modified Flanker Task)/48 h/08:00–10:00 h	Familiarization trials of all physical tests completed on trial day/**CAFF (coffee) vs. PLAC (decaf coffee)/**crossover, counterbalanced, randomized/double-blind	“Full stomach”/144.00 ± 113.36 mg and 207.78 ± 130.14 mg/3 mg·kg^−1^, 60–75 min prior	Not given/Takei digital handgrip dynamometer, Takei digital back strength dynamometer/modified Flanker (reaction time)	Not given/not given	Not given/not given/not given	**Handgrip:** CAFF vs. PLAC *p* < 0.001 (Δ% not reported) **Back strength:** CAFF vs. PLAC *p* = 0.007 (Δ% not reported)
Souissi et al. [11]	15/male/20 ± 1 y/a priori = 14	Physically active/NA/NA	22:00 h to 06:00 h/not given/intermediate type	Attention (number of correct responses) and reactions time (RT)/≥3 days between test sessions/07:00, 09:00 h	Two sessions/PLAC vs. CAF (07:00 and 09:00)/randomized, balanced crossover; double-blinded	Standardized meal/no caffeine during testing weeks/6 mg·kg^−1^ 60 min prior	NA/NA/software “React” for simple reaction time; attention assessed with numbers cancellation test	Not given/not given	28.2–29.3 °C/45.1–46.7%/not given	**Reaction time 07:00 h:** CAF vs. PLAC Δ6.4% *p* < 0.05 **Reaction time 09:00 h:** CAF vs. PLAC Δ4.1% *p* < 0.05 **Attention (correct responses) 07:00 h:** CAF vs. PLAC Δ2.9% *p* < 0.05 **Attention (correct responses) 09:00 h:** CAF vs. PLAC Δ1.5% *p* < 0.05
Wilk et al. [58]	15/male/26.8 ± 6.2 years/not given	Minimum 3 years of strength training experience/1RM BP = 122.3 ± 24.5 kg	Not given/not given/not given	BP with LE/7 days/09:00–11:00 h	One session/PLAC vs. CAFF (3 mg) vs. CAFF (6 mg) vs. CAFF (9 mg)/randomized, crossover/double-blinded	Not given/426 ± 102 mg/caffeine: 3, 6, 9 mg·kg^−1^ 60 min prior	Not given/Tendo Power Analyzer)/not applicable	Not given/not given	Not given/not given/not given	No significance
Mora-Rodriguez et al. [13]	13/male/21.9 ± 2.9 years/not given	Resistance trained (7.1 ± 3.5 years)/1RM BS = 112.5 ± 12.6 kg, BP = 121.0 ± 22.7 kg	Not given/not given/not given	Smith machine BP and BS with LE/36–48 h/08:00 h	Seven session s/CAFF vs. PLAC/double-blinded, cross-over, randomized, placebo-controlled	Standardized diet (breakfast 60 min prior)/light caffeine consumers (≤70 mg.day^−1^)/6 mg·kg^−1^ 60-min prior	Tympanic temperature/Smith machines (Multipower Fitness Line, Peroga, Spain), T-Force System linear velocity transducers (Ergotech)/not applicable	Not given/not given	Not given/not given/not given	**BS (MPV):** CAFF vs. PLAC Δ5.4–8.1%; *p* <0.05 (for 25%, 50%, 75% 1RM)
Pallarés et al. [59]	13/male/21.9 ± 2.9 years/not given	Resistance-trained (7.1 ± 3.5 years)/1RM BS = 112.5 ± 12.6 kg; BP = 121.0 ± 22.7 kg	Not given/not given/not given	Smith machine BP and BS with LE/48 h/08:00 h	Seven sessions/PLAC vs. CAFF 3 mg vs. CAFF 6 mg vs. CAFF 9 mg/randomized, double-blind, crossover, placebo-controlled	Standardized diet (breakfast 60 min prior)/light caffeine consumers (≤ 70 mg day^−1^)/6 mg·kg^−1^ 60-min prior	Tympanic temperature/Smith machines (Multipower Fitness Line, Peroga, Spain), T-Force System linear velocity transducers (Ergotech)/not applicable	Not given/not given	Not given/not given/not given	BP and BS (MPV): **25–50% 1RM:** CAFF 3/6/9 vs. PLAC Δ5.4–8.5% *p* = 0.039–0.000 **75% 1RM:** CAFF 6/9 vs. PLAC Δ6.3–8.9% *p* = 0.046–0.014 **BP (MPV):** 90% 1RM: CAFF 9 vs. PLAC Δ13.1% *p* = 0.031 **BS (MPV):** 90% 1RM: CAFF 9 vs. PLAC Δ10.4% *p* = 0.046; CAFF 6 vs. PLAC Δ8.3% *p* = 0.029 BP and BS (MPP): **25–50% 1RM**: CAFF 3/6/9 vs. PLAC Δ8.1–12.0% *p* = 0.022–0.000 (except CAFF 3 in BS) **75% 1RM:** CAFF 6/9 vs. PLAC Δ8.3–10.2% *p* = 0.037–0.010 **90% 1RM:** CAFF 9 vs. PLAC Δ11.7–15.0% *p* = 0.031–0.014; CAFF 6 in BP Δ11.4% *p* = 0.021
Soussi et al. [11]	12/not given/21.08 ± 1.16 years/not given	Elite Judoists (judokas)/not applicable/not applicable	23:00 ± 00:30 h to 06:30 ± 00:30 h/not given/no “extreme type”	Simple reaction time/48 h/06:00–07:00 h	“Several” sessions/CAFF vs. PLAC/not given	Not given/reported non-consumers of caffeine/5 mg·kg^−1^ caffeine (coffee) ingested 60 min prior	NA/NA/software “React” for simple reaction time	Not given/not given	Not given/not given/not given	**Simple reaction time:** CAFF vs. PLAC Δ11.8 *p* < 0.001
Mora-Rodriguez et al. [9]	12/male/19.7 ± 2.8 years/not given	Resistance-trained (7.2 ± 2.4 years); BP and BS 1RM reported relative to body mass (BP = 1.15 ± 0.08; BS = 1.46 ± 0.15); light caffeine users (≤60 mg.day^−1^)	Not given/not given/not given	BS and BP bar-velocity testing (1 ms^−1^ load and 75% 1RM), MVCLEG, EVOKLEG, handgrip/trials separated by 24–36 h/10:00 h	Three sessions/CAFF vs. PLAC/randomized double-blind crossover placebo-controlled design	Standardized in-residence diet; abstained alcohol/tobacco/caffeine 4 days pre-testing/caffeine 3 mg·kg^−1^ in capsules with standardized meal 60 min pre-testing	Tympanic temperature (Braun Thermoscan); T-Force linear encoder; Tedea Huntleigh 1263 and electrical stimulator; cognitive tests not applicable	Not given/not given	Thermoneutral; 19 °C dry bulb/not given/not given	**EVOKEDLEG:** Peak evoked twitch CAFF vs. PLAC Δ16% *p* = 0.05 (*n* = 7) **BS velocity (75% 1RM):** CAFF vs. PLAC Δ5.3% *p* = 0.003–0.023. **BP velocity (75% 1RM):** CAFF vs. PLAC Δ4.6% *p* = 0.003–0.023. **BS velocity (~1.00 m/s load):** CAFF vs. PLAC Δ2.5–7.5% *p* = 0.000–0.002
Maridakis et al. [52]	18/male/22.7 ± 4.0/a priori = 18	NA	Not given/not given/not given	Battery of cognitive tests/≥24 h/within 90 min of usual wake time	Four conditions, PLAC, 100 mg or 200 mg doses, 440 kcal breakfast/counterbalanced	8 h fasted (not including breakfast condition)/61.2 ± 44.4 mg.day^−1^/100 mg, 200 mg or PLAC ~135 min prior	NA/sustained attention (vigilance), motor (finger) response, Bakan Test.	Not given/not given	Not given/not given/not given	100 mg and 200 mg of caffeine vs. placebo produced **small–moderate** improvements **in vigilance performance: reduced reaction times, improved target identification** and **fewer false alarms on Bakan primary and secondary tasks (no Δ% reported; effects described via Hedges’ d; all *p* < 0.05 for main cognitive effects).**
Smith et al. [60]	48/24 males, 24 females/not given/not given	NA	Not given/not given/Morningness–Eveningness Questionnaire completed but chronotype categories not reported	Battery of cognitive tests/NA/07:45 baseline, 11:30 h pre-lunch	Not given/breakfast + CAF, breakfast + DECAF + no breakfast + CAF, no breakfast + DECAF/between-subject design, group allocation method not specified/double-blinded	Fasting before AM/typically 2 cups of coffee per day/4 mg·kg^−1^ at 08:45 and 11:15 h	NA/cognitive tests: free recall (20-word list), delayed recognition memory (40 words), semantic processing (true/false sentences), logical reasoning (Baddeley letter-order task), repeated digits vigilance (3-digit numbers at 100/min; hits, reaction time, false alarms)	Not given/not given	Not given/not given/not given	**Logical reasoning:** CAF vs. DECAF % correct Δ9.0% *p* < 0.005; RT per sentence Δ15.2% *p* < 0.05 **Semantic processing:** CAF vs. DECAF Δ7.6% *p* < 0.05 **Repeated digits vigilance:** CAF vs. DECAF hits Δ50.5% *p* < 0.0001; RT Δ13.3% *p* < 0.0001
Mitchell and Redman [8]	19/13 female and 6 male/19–39 years/not given	NA	Not given/not given/not given	Five cognitive “Academic type tasks”/7 days/07:00 h	One session/CAFF vs. PLAC/counterbalanced/not given	No CAFF/psychoactive 24 h prior, no food and smoking 1 h prior/*n* = 6 <120 mg, *n* = 8 >120 mg <300 mg and *n* = 8 > 300 mg.day^−1^/4 mg·kg^−1^ body mass 60 min before session	NA/NA/20-item short-term memory (STM) task, mental arithmetic (MA), reading comprehension, serial search (SS) and verbal reasoning (VR) tasks	Not given/not given	Not given/not given/not given	**Serial search time:** CAFF vs. PLAC Δ2.5%; (*p* < 0.01). **Number correct:** PLAC vs. CAFF (*p* < 0.002) **VR time taken:** CAFF vs. PLAC Δ6.3% *p* < 0.01

Lastly, the current study supports the proposed mechanism of action of caffeine, which is attributed to antagonism of adenosine receptors within the central nervous system, leading to reduced perception of effort, fatigue resistance and enhanced central motor drive. Our and other findings (Table 5) demonstrate that caffeine may have applied value in early-morning training, where baseline neural readiness and force-generating capacity are otherwise suppressed. However, the influence of caffeine on post-exercise-recovery is less clear, with a recent systematic review showing no consistent improvement in indirect markers of exercise-induced muscle damage or inflammation due to the low number of studies (four studies) and controversial results for both dynamic and isometric strength tests [6,72]. There is a possibility that caffeine’s primary recovery-related benefits may be perceptual and neural rather than structural or biochemical in nature. This comes from limited evidence suggesting caffeine may improve subjective recovery, reduce perceived soreness and accelerate recovery of certain neuromuscular function indices following fatiguing exercise [6,73]. Examining whether caffeine not only enhances morning resistance exercise performance but also meaningfully alters the subsequent fatigue–recovery profile following such sessions has practical importance, especially for athletes and practitioners who rely on early-morning training and may use caffeine both to enhance session quality and potentially influence recovery kinetics [74]. This provides a strong rationale to examine the effects of caffeine on both morning strength and fatigue development and the subsequent time-course of neuromuscular and perceptual recovery following resistance exercise.

## 5. Limitations

Only young male participants volunteered for the current study, despite opening recruitment to both sexes. We acknowledge that more female-specific work of between-sex comparisons is needed as biological sex may be a potential factor modulating the effect of caffeine. Participants were recruited from undergraduate and postgraduate students; hence the age ranges were narrow (22.4 ± 3.8 years old, Table 1). We did not measure caffeine status in the blood from ingestion to the end of protocol, which would have enabled individual pharma-kinetics to better quantify caffeine and performance. We recruited intermediate types and caffeine-naive participants, meaning our findings may not be transferable to extreme chronotypes, females, elite populations or high habitual caffeine daily users [58,59]. We acknowledge that including a group with high daily caffeine consumption would have added another dimension to the work. The significant effect of the displacement variable for both bench press and back squat by load indicates that ‘form’ was lost as the load on the bar increased, despite participant familiarization sessions. Therefore, future studies should consider recruiting individuals with a higher ‘resistance training age’ or reflect on how form can be retained such that it does not affect other Muscle Lab linear encoder variables, and the lift can be ‘standardized’. Lastly, we did not measure caffeine levels every 15 min from consumption of the pills to the end of the study, nor did we measure a marker of genetic and epigenetic sensitivity (such as CYP1A2 or possibly ADORA2A), which are associated with caffeine metabolism, sensitivity and response.

## 6. Conclusions

Our most important outcome was that CAFF in low caffeine consumers improved morning isometric peak isometric force in NoPill and PLAC conditions (with stimulation, Δ8.7 and 9.0%). Caffeine improved peak isometric force without stimulation in the NoPill condition (Δ8.3% with a large effect size of η^2^_p_ = 0.42). Average and mean propulsive velocity in bench press (Δ3.3 and 4.6%) and back squat (Δ7.7 and 9.2%) were higher in CAFF compared to the NoPill and PLAC conditions. These findings confirm our first hypothesis in individuals who followed a standardized protocol that incorporated some of the main concerns in the literature regarding chronobiological study design. A second aim of the current study was to investigate the effect of caffeine ingestion on morning cognitive performance (including tasks of attention, memory, and executive function). We found caffeine selectively improved short-term verbal recall, without consistent effects on executive or attentional performance compared to NoPill, but no difference between CAFF and PLAC values was observed. Inclusion of the NoPill condition allowed a placebo effect to be considered, with success achieved in disguising the caffeine, hence reducing the nocebo effect (bias due to thinking you were on the PLAC condition, negatively influencing your performance).

## 7. Practical Implications and Future Research

To our knowledge, this is the only study to investigate isokinetic MVC force outputs and submaximal weightlifting performance relating to CAFF ingestion where a control for the placebo effect has been employed. The current findings provide important recommendations and interventions for athletes who have high training/competition demands. Based on our results, the effectiveness of acute (1–1.5 h) CAFF supplementation in a healthy population who are not high habitual users of CAFF is strong. In the current study, the observed large effect size (η^2^_p_ = 0.31 and 0.42) of acute caffeine supplementation increasing morning isometric MVC force production has practical as well as physiological significance, where the observed improvements (~10%) are similar to levels observed by others in the evening (10–15%) [2,3], giving some evidence that caffeine may offset the diurnal variation in this measure. Because velocity-based training (VBT) metrics have very low day-to-day noise, even small changes can be meaningful. But the threshold depends on (i) lift, (ii) load, (ii) device reliability and (iv) training status of participants [75,76]. The current finding of a 3–5% increase in AV/MPV generally sits at or just above the typical measurement error of high-quality velocity-tracking devices, which often show SEM values of ~0.01–0.09 m·s^−1^ and CV < 8–9% [75,77]. Because this range overlaps with the smallest worthwhile change, such improvements may reflect acute neuromuscular readiness rather than meaningful chronic adaptation. While velocity metrics are sensitive indicators of day-to-day neuromuscular status, larger changes (≥8–10%) are more consistently associated with real training adaptation and functional performance improvement [78,79]. Thus, a 3–5% rise is borderline meaningful and should be interpreted cautiously and confirmed across repeated sessions. Further work should investigate the interindividual response to caffeine in the cognitive and strength measures, in male and female populations, as well as habitually low, medium and high caffeine consumers, which could increase the validity of our findings. Venous blood sampling would be integral to establishing both post-supplement CAFF serum levels and ‘pre’-habitual CAFF status. Average velocity and average propulsive velocity are used in velocity-based training; despite being a relatively underexplored approach for monitoring resistance training sessions, the current findings of improvements with caffeine ingestion warrant further investigation. Lastly, we propose exploring the effects of caffeine on both morning strength and fatigue development and the subsequent time-course of neuromuscular and perceptual recovery following resistance exercise.

## Figures and Tables

**Table 1:** Physical characteristics and habitual daily intake of the participants (mean ± SD values).

Physical Characteristics	Participant Variables	
Age (year)	22.4 ± 3.8	
Height (cm)	176.2 ± 6.7	
Mass (kg)	83.9 ± 15.7	
Chronotype	33.0 ± 5.9	
Languid/vigorous	43.5 ± 6.0	
Flex/rigidity	45.3 ± 8.6	
Athletic level (1–5)	1.7 ± 0.9	
Habitual sleep (decimal h)	9.3 4.1	
1RM bench press (kg)	92.5 ± 18.8	
1RM back squat (kg)	122.0 ± 23.0	
Daily caffeine intake from questionnaire (mg)	110.0 ± 45.1	
**Habitual Daily Intake**	**Participants’ Intake**	RDI *
Calories (kcal)	2121 ± 518	2500 (*p* = 0.101)
Fats (g)	88 ± 36	97 (*p* = 0.519)
Protein (g)	**154 ± 44**	**55.5 (*p* < 0.001)**
Carbohydrates (g)	**179 ± 55**	**333 (*p* < 0.001)**
Zinc (mg)	11.4 ± 3.4	9.5 (*p* = 0.456)
Magnesium (mg)	275 ± 103	300 (*p* = 0.672)
Vitamin B6 (mg)	2.5 ± 0.9	1.6 (*p* = 0.222)
Caffeine (mg)	**37.3** ± **37.6**	NA
Water intake (mL)	**3001** ± **1197**	**2000 (*p* = 0.069)**

* Denotes recommended daily intake values (RDI) from the Nutrition Science Team [21]. **Bold** values denote significant participant values compared to RDA from a paired *t*-test. NA indicates no recommended daily intake values.

**Table 2:** Mean ± SD values for main effect for temperatures and all performance variables measured in this study in the three experimental conditions: no-pill control (NoPill), placebo (PLAC) and caffeine ingestion (CAFF). *p* values, partial eta squared values (η^2^_p_) and observed power (OP) are given.

Variable	NoPill	PLAC	CAFF	Significance Condition (*p*, η^2^_p_, OP)	Significance of Load/Pre-Post (*p*, η^2^_p_, OP)	Interactions (*p*, η^2^_p_, OP)
**Temperature**						
Rectal temperature (°C)	37.04 ± 9.39	37.01 ± 0.64	36.98 ± 9.39	*p* = 0.883, 0.006, 59.0	***p*** **= 0.012, 0.40, 77.4**	*p* = 0.873, 0.008, 6.4
Mean skin temperature (°C)	30.12 ± 7.80	29.30 ± 7.91	30.61 ± 7.91	*p* = 0.078, 0.18, 50.4	***p*** **< 0.001, 0.86, 100**	*p* = 0.280, 0.093, 26.0
Mean body temperature (°C)	34.55 ± 0.67	34.24 ± 0.58	34.24 ± 0.40	*p* = 0.109, 0.16, 44.3	***p*** **< 0.001, 0.78, 100**	*p* = 0.447, 0.060, 17.7
**Isokinetic (MVC)**						
MVC peak force stimulation (N)	**1054 ± 213**	**1057 ± 201**	1158 ± 292 *^#^	***p*** **= 0.008, 0.31, 83.5**		
% Activation	**86.8 ± 7.6**	**84.2 ± 8.5**	91.1 ± 6.5 *^#^	***p*** **= 0.016, 0.33, 74.0**		
RPE (6–20)	18.4 ± 1.8	18.8 ± 1.6	18.0 ± 2.3	*p* = 0.178, 0.13, 32.1		
RPE (0–10)	8.9 ± 1.1	9.3 ± 0.9	9.0 ± 1.0	*p* = 0.282, 0.09, 26.1		
Exertion (1–10)	9.1 ± 1.1	9.2 ± 0.8	8.9 ± 1.1	*p* = 0.423, 0.06, 16.6		
MVC peak force no-stim (N)	**1080 ± 204**	**1111 ± 195**	**1178 ± 228 ^#^**	***p*** **= 0.001, 0.42, 94.9**		
RPE general (6–20)	**17.8 ± 2.4**	**17.7 ± 2.1**	16.9 ± 3.0 *^#^	***p*** **= 0.040, 0.25, 58.8**		
RPE effort (0–10)	8.4 ± 2.0	8.5 ± 1.7	8.2 ± 1.9	*p* = 0.500, 0.05, 14.9		
Exertion in task (1–10)	8.9 ± 1.4	8.4 ± 1.2	8.5 ± 1.5	*p* = 0.218, 0.11, 31.1		
**Bench Press**						
Average power (W)	413 ± 113	405 ± 109	427 ± 116	*p* = 0.062, 0.20, 54.2	***p*** **< 0.001, 0.75, 100**	*p* = 0.905, 0.02, 10.1
Displacement (cm)	45.9 ± 5.9	45.6 ± 5.7	45.9 ± 3.1	*p* = 0.672, 0.28, 10.1	***p*** **< 0.001, 0.74. 100**	*p* = 0.909, 0.02, 100
Average velocity (ms^−1^)	**0.75 ± 0.28**	**0.74 ± 0.28**	0.77 ± 0.28 *^#^	***p*** **= 0.031, 0.24, 66.2**	***p*** **< 0.001, 0.98, 100**	*p* = 0.751, 0.04, 14.8
Peak velocity (ms^−1^)	1.15 ± 0.43	1.14 ± 0.44	1.15 ± 0.45	*p* = 0.772, 0.01, 6.5	***p*** **< 0.001, 0.98, 100**	*p* = 0.676, 0.04, 13.1
Time to peak velocity (s)	0.52 ± 0.26	0.53 ± 0.26	0.52 ± 0.22	*p* = 0.709, 0.02, 8.2	***p*** **< 0.001, 0.79, 100**	*p* = 0.865, 0.01, 6.2
Mean propulsive velocity (ms^−1^)	**0.78 ± 0.31**	**0.77 ± 0.31**	0.82 ± 0.32 *^#^	***p*** **= 0.037, 0.24, 61.7**	***p*** **< 0.001, 0.97, 100**	*p* = 0.906, 0.01, 8.4
RDV (ms^−2^)	2.98 ± 1.96	2.79 ± 1.86	2.88 ± 1.90	*p* = 0.272, 0.10, 22.1	***p*** **< 0.001, 0.96, 100**	*p* = 0.590, 0.04, 14.4
RPE general (6–20)	12.6 ± 3.1	11.9 ± 4.2	12.5 ± 3.1	*p* = 0.583, 0.04, 11.9	***p*** **< 0.001, 0.93, 100**	*p* = 0.918, 0.02, 9.7
RPE effort (0–10)	7.7 ± 2.1	7.9 ± 2.4	7.9 ± 2.5	*p* = 0.503, 0.05, 12.9	***p*** **< 0.001, 0.95, 100**	*p* = 0.075, 0.15, 62.0
Exertion in task (1–10)	9.0 ± 2.0	9.2 ± 2.2	9.2 ± 2.1	*p* = 0.452, 0.56, 15.6	***p*** **< 0.001, 0.89, 100**	*p* = 0.240, 0.10, 31.0
**Back Squat**						
Average power (W)	1109 ± 380	1261 ± 345	1219 ± 410	*p* = 0.104, 0.18, 40.3	***p*** **< 0.001, 0.74, 100**	*p* = 0.249, 0.10, 25.0
Displacement (cm)	**61.7 ± 7.3**	**61.7 ± 7.4**	64.3 ± 7.0 *^#^	***p*** **= 0.015, 0.28, 75.8**	***p* <** **0.001, 0.56, 98.2**	*p* = 0.239, 0.10, 41.2
Average velocity (ms^−1^)	**0.73 ± 0.19**	**0.76 ± 0.18**	0.79 ± 0.18 *	***p*** **= 0.031, 0.23, 66.2**	***p*** **< 0.001, 0.98, 100**	*p* = 0.751, 0.04, 14.8
Peak velocity (ms^−1^)	1.15 ± 0.43	1.14 ± 0.44	1.15 ± 0.45	*p* = 0.772, 0.01, 6.5	***p*** **< 0.001, 0.98, 100**	*p* = 0.676, 0.04, 13.1
Time to peak velocity (s)	0.70 ± 0.26	0.68 ± 0.25	0.74 ± 0.28	*p* = 0.311, 0.08, 17.9	***p*** **< 0.001, 0.74, 100**	*p* = 0.299, 0.89, 25.7
Mean propulsive velocity (ms^−1^)	**0.76 ± 0.21**	**0.79 ± 0.22**	0.84 ± 0.22 *	***p*** **= 0.015, 0.32, 74.6**	***p*** **< 0.001, 0.96, 100**	***p*** **= 0.037, 0.21, 66.0**
RDV (ms^−2^)	2.26 ± 0.99	2.39 ± 1.11	2.45 ± 1.05	*p* = 0.116, 0.16, 40.9	***p*** **< 0.001, 0.94, 100**	***p*** **= 0.013, 0.24, 80.5**
RPE general (6–20)	13.3 ± 3.0	13.3 ± 3.4	13.3 ± 3.2	*p* = 0.779, 0.01, 6.3	***p*** **< 0.001, 0.91, 100**	*p* = 0.295, 0.01, 26.3
RPE effort (0–10)	4.6 ± 2.1	5.0 ± 2.2	4.4 ± 2.3	*p* = 0.224, 0.11, 29.6	***p*** **< 0.001, 0.93, 100**	*p* = 0.200, 0.11, 36.3
Exertion in task (1–10)	6.0 ± 2.0	6.5 ± 2.0	6.1 ± 1.9	*p* = 0.185, 0.12, 32.8	***p*** **< 0.001, 0.81, 100**	*p* = 0.601, 0.04, 14.4

**Bold** values are statistically significant (*p* < 0.05); underlined values indicate a trend (0.1 < *p* > 0.05). * indicates > or <NoPill; ^#^ indicates > or <PLAC conditions.

**Table 3:** Mean ± SD values for main effect for cognitive performance tests (‘trail-making [A, B]’, ‘Rey’s auditory’ and ‘Stroop’) in the three experimental conditions (no-pill control (NoPill), placebo (PLAC) and caffeine ingestion (CAFF)). *p* values, partial eta squared values (η^2^_p_) and observed power (OP) are given.

Variable	NoPill	PLAC	CAFF	Significance Condition (*p*, η^2^_p_, OP)
**Cognitive tests**				
Trail-making test A (s)	13.4 ± 3.0	13.5 ± 2.9	13.7 ± 2.9	*p* = 0.954, 0.004, 5.6
Trail-making test B (s)	28.7 ± 6.6	25.7 ± 5.8	29.0 ± 13.4	*p* = 0.436, 0.052, 12.9
Rey’s auditory—total number	**51.9 ± 13.1**	**56.3 ± 10.3**	56.8 ± 11.6 *	***p* = 0.040, 0.22, 61.3**
Rey’s auditory—distractions	7.2 ± 2.8	6.4 ± 2.1	6.9 ± 2.5	*p* = 0.599, 0.04, 12.7
Rey’s auditory—retention	10.1 ± 3.4	10.9 ± 2.8	10.9 ± 3.5	*p* = 0.472, 0.06, 16.7
**Stroop**				
Colours—number	64.5 ± 12.1	66.6 ± 12.4	62.3 ± 10.9	*p* = 0.141, 0.142, 38.2
Colours—error	1.5 ± 1.3	1.4 ± 1.2	1.1 ± 1.2	*p* = 0.716, 0.025, 9.8
Colours interference—number	15.6 ± 7.3	20.3 ± 6.8	16.3 ± 5.6	*p * = 0.084, 0.162, 49.3
Word—number	114.1 ± 21.8	118.6 ± 23.8	113.8 ± 25.5	*p* = 0.404, 0.063, 19.5
Word—error	1.1 ± 1.1	0.8 ± 1.0	1.0 ± 1.2	*p* = 0.634, 0.027, 9.6
Colours interference—error	9.2 ± 6.7	6.5 ± 4.3	8.6 ± 4.8	*p* = 0.368, 0.069, 21.2

**Bold** values are statistically significant (*p* < 0.05); underlined values indicate a trend (0.1 < *p* > 0.05). * indicates > or <NoPill.

**Table 4:** Mean (±SD) values for sleep questions, tiredness and alertness, profile of mood states (POMS) and caffeine withdrawal symptom scores for three conditions (NoPill, PLAC and CAFF), with statistics given [*p* values, partial eta squared values (η^2^_p_) and observed power (OP)].

Variables	NoPill	PLAC	CAFF	Significance Condition
**Sleep questions**				
Get to sleep	0.00 ± 1.75	−0.07 ± 2.13	0.07 ± 1.86	*p* = 0.981, 0.001, 5.3
Well Slept	1.64 ± 1.91	0.64 ± 1.60	1.29 ± 2.20	*p* = 0.371, 0.072, 19.4
Waking time	−1.50 ± 1.99	−2.07 ± 1.73	−1.57 ± 165	*p* = 0.708, 0.024, 9.4
Alertness after waking	0.64 ± 1.34	−0.07 ± 1.77	0.64 ± 1.65	*p* = 0.354, 0.077, 21.9
Tiredness (0–10 VAS)	4.1 ± 2.7	3.7 ± 2.4	4.0 ± 2.5	*p* = 0.807, 0.015, 7.5
Alertness (0–10 VAS)	6.1 ± 1.8	6.1 ± 2.1	5.8 ± 2.3	*p* = 0.636, 0.034, 11.7
**POMS**				
Mood State—Vigour	7.6 ± 3.6	7.4 ± 3.7	7.6 ± 4.4	*p* = 0.979, 0.002, 5.3
Mood State—Anger	1.0 ± 1.8	1.0 ± 2.3	0.6 ± 0.9	*p* = 0.655, 0.030, 10.5
Mood State—Tension	0.8 ± 1.3	0.6 ± 1.2	0.4 ± 0.8	*p* = 0.533, 0.038, 10.9
Mood State—Calm	8.4 ± 3.2	8.6 ± 2.4	7.9 ± 3.5	*p* = 0.678, 0.027, 9.9
Mood State—Happiness	7.4 ± 2.9	7.6 ± 2.7	7.5 ± 3.2	*p* = 0.986, 0.001, 5.2
Mood State—Confusion	0.7 ± 1.3	0.6 ± 1.2	0.5 ± 0.9	*p* = 0.841, 0.012, 7.1
Mood State—Depression	0.7 ± 1.5	0.7 ± 1.5	0.1 ± 0.5	*p* = 0.244, 0.104, 23.8
Mood State—Fatigue	8.8 ± 3.8	8.7 ± 3.8	8.3 ± 5.3	*p* = 0.261, 0.098, 26.6
**Caffeine Withdrawal symptom scores**	25.6 ± 8.5	24.3 ± 6.7	24.8 ± 12.7	*p* = 0.845, 0.010, 6.7

## Data Availability

The data presented in this study are available on request from the corresponding author due to privacy or ethical restrictions.
